# Bibliographical Notices

**Published:** 1844-08-24

**Authors:** 


### BIBLIOGRAPHICAL NOTICES.

*A Practical Treatise on Midwifery: Exhibiting the present advanced State of the Science.* By F. J. Moreau, Officer of the Legion of Honour and of the Order of Leopold; Professor of Midwifery and the diseases of women and children in the Faculty of Medicine of Paris; Physician to the Lying-in Hospital of Paris; Consulting Surgeon to the King, &c. &c.
*Translated from the French,* by Thomas Forrest Betton, M. D., and Edited by Paul B. Goddard, A. M., M. D., Lecturer on Anatomy and Demonstrator in the University of Pennsylvania, &c. &c. *With Eighty plates, comprising numerous separate illustrations.* 4to., pp. 235. Philadelphia, Carey and Hart, for G. N Loomis, 1844.

The first thing that takes our attention on looking at this valuable publication, is its admirable mechanical execution. The text is beautifully printed on fine white paper, and the illustrations are deserving of all praise. When we look back a very few years, and contrast the labours of our artists then with such specimens as the present, we are astonished at the great improvement which has been achieved in so brief a space of time. For the lithographed plates in the volume before us, we are, we believe, altogether indebted to Philadelphia artists, and the liberality of the very respectable publishers of the work.

The profession in this country are under great obligations to the Translator and Editor for enabling them to read the work of M. Moreau in their own language; for notwithstanding we have so many excellent treatises on the same subject, some of them, too, amply illustrated, still there is always room for one like the present. The author is not a man of yesterday,—no stripling,—but one of large experience and deep reflection,—long one of the brightest ornaments of the profession in the French Metropolis. We find in his work no silly affectation of originality, but a frank avowal of the various authorities from whom he derives important facts and conclusions. In the bibliographical part,—which, although less copious than in several more recent productions, especially English and German, exhibits nevertheless considerable research,—the author acknowledges the assistance of the accomplished Brierre de Boismont.

The text treats of the usual subjects embraced in works on Midwifery, commencing with an account of the anatomical structure and peculiarities of the female pelvis and organs of generation, and their abnormal deviations; their functions in health, and the interruption of these by disease, together with the treatment to be pursued. Next comes conception, with an account of the ovum, and the signs of pregnancy. On the latter subject, although the symptoms are treated in detail, some of the indications pointed out by modern observation are not included. The principal part of the work, however, is devoted to the consideration of labour: commencing with "*natural labours,*" or such as terminate in a reasonable time without assistance, and extending through all the complications and difficulties that arise. The whole are divided into *Classes, Orders, Genera, Species,* and *Varieties,* constituting the most artificial, complex, and difficult scheme for a young beginner, that we have met with in any late work on the subject. We regret this exceedingly; for however ingenious such elaborate classifications, with their divisions and subdivisions, may appear to authors, students rarely comprehend them, and practitioners never heed them in their daily pursuits. Some such arrangement is proper and necessary, but the simpler the classification and the fewer the subdivisions, so that all essential points of practice are embraced, the more likely is the subject to be investigated and understood by the student, and all beyond that, only perplexes and disgusts him. The practice inculcated by Professor Moreau, is on every point, so far as we have observed, judicious, and in many instances, illustrated by the narration of cases so apposite and so clearly and concisely told, that the dullest intellect can hardly misapprehend or forget what the author aims to inculcate.

The work of Professor Moreau is a treasure of obstetrical science and practice, and the American Edition of it, an elegant specimen of the arts.

*On Superstitions connected with the history and practice of Medicine and Surgery.* By Thomas Joseph Pettigrew, F. R. S., F. S. A., Doctor of Philosophy of the University of Göttingen, Surgeon to H. R. H., the Duchess of Kent, &c. &c. 12mo., pp. 213. Philadelphia, Ed. Barrington and Geo. D. Haswell, 1844.

This is a reprint of the London Edition, published last year. It is not only a neat little volume, well got up, but a most amusing, and in some respects, instructive one, which will well repay a perusal. It exhibits a sorry picture of the craft and weakness of the human mind, it is true; nevertheless, by the contrast which it affords when compared with the fruits of science and philosophy at the present day, we are enabled to form a better conception of the mighty powers with which the human intellect is endowed. At any rate, from the follies of the past we may derive wisdom for the future, and there is much in the volume before us that may be profitably used for this purpose.

*Notes on Ovariotomy.* By Fleetwood Churchill, M. D., M. R. I. A., Hon. Fellow of the Philadelphia Medical Society; Physician to the Western Lying-in-Hospital and Dispensary; and Lecturer on Midwifery, &c., at the Richmond School of Medicine." (Read before the Dublin Obstetrical Society.)

(For this and two other interesting communications, which we shall notice hereafter, we are indebted to the kindness of Dr. Churchill, and beg he will accept our thanks for the same.)

In this paper we have, condensed into a small compass, the whole of our experience in regard to this fearful operation. After noticing "one or two points in the pathology and history of the disease for which ovariotomy is proposed as a remedy," the author gives a brief account of the cases operated on, the names of the operators, the time when the operation was performed, and the results; and for more convenient reference, the whole is then thrown into tables.

The following is the concluding part of this able paper:

Let us now attempt a little closer analysis of these cases. It will be remembered that the question at present is not whether each operation was justifiable or suitable, but merely as to the results of the operation under given circumstances.

1. The entire number of cases—whether dropsy, or scirrhus of the ovary, uterine disease, or simulated tumours—amount to sixty-six: of these forty-two recovered, and twenty-four died, or about 1 in 2¾.

Of the forty-nine cases in which the ovary was extirpated, sixteen died, or 1 in 3 1-16ths. Of the nine cases in which the operation could not be completed, four died, or 1 in 2¼; and of the eight cases where the operation was unnecessary, four died, or 1 in 2.

2. It is not quite so easy to give the comparative mortality of the long and short incision, because the definition of each is scarcely settled. Taking the length of the wound as our guide, without reference to the tapping of the tumour before extraction, we will include all cases under the term "minor operation," where the incision did not exceed four inches; and under the term "major operation," where it exceeded that.

Of the true ovarian cases in table 1, there are fourteen cases of the short operation, of these thirteen recovered, and two died; and thirty-four cases of the long operation, of whom twenty-one recovered, and thirteen died, or 1 in 2 8-13ths. In the second and third table there are fifteen cases of the long operation, of whom seven died, or 1 in 2 1-7th. Of the forty-nine cases of the long operation twenty died, or 1 in 2½. At the same time it must be observed that in the cases of the short operation there are much less irritation and injury owing to the absence of adhesions; and in some cases the short operation would have been perfectly useless, so that if any attempt were to be made, it must be by the long incision, with all its risks. The comparison therefore is not quite fair.

3. Age does not appear to have had much influence upon recovery or death, for the ages mentioned in six of the fatal ovarian cases were 23, 25, 40, 40, 47, and 59; whilst those of the successful ones range between 20 and 60.

The same may be said, as far as the information extends, of the married or single condition of the patients.

4. At first sight one would expect a considerable variation in the result of cases in which there were adhesions from those in which there were none, because of the violence necessary; and this seems to be confirmed by the cases of Chrysmer and others, where the patients died of gangrene of the peritoneum; yet of seventeen cases in which the adhesions were found, and in some very extensive, eleven recovered, and six died. This, however, shows the great disadvantage of adhesions, and there are certain cases, one of which we have just seen, in which these were so extensive that removal of the tumour would have been impossible.

5. Certain of the operations (Chrysmer, Clay, &c.) were performed upon women labouring under other organic diseases, or suffering from great constitutional exhaustion, and these cases proved fatal.

6. The operation was several times frustrated by the excessive vascularity of the tumour, or its firm attachment to the pelvis, and though several of them (four out of eight) recovered, yet these are additional reasons for serious investigation.

7. It is further shown by table 3, that the operation was performed when no tumour at all existed—when the tumour was uterine, or growing from the pelvis, or an hydatid. At first sight it might be supposed that such errors of diagnosis were the result of carelessness, and that the first could scarcely occur. And yet Mr. Lizars is a surgeon of no mean experience; and M. Dolhoff had his patient in hospital under his observation for a year or so. Very lately I was consulted for a supposed ovarian tumour, and upon examination there was a distinctly shaped abdominal tumefaction, which had all the feel of a uterine or ovarian tumour, and yet upon calling off the patient's attention, and setting the abdominal muscles into action, it entirely vanished.

It may be worth while now to look a little closer at the *diagnosis* of these tumours.

1. The abdominal muscles appear to acquire the power of involuntarily assuming the form and appearance, and of communicating the sensation of a tumour. In some cases it seems as though the result of the form given to them by a former pregnancy. Against this deception we can in a great measure guard ourselves, by prolonging our abdominal manipulation, and calling the muscles into action by leading the patient to converse. Percussion will also aid us in coming to a right conclusion, and if we make an examination per vaginam and per rectum, there will be but little doubt remaining. And I would observe that an examination per rectum is most valuable in all cases of real or supposed ovarian disease.

2. In the majority of cases the continuity of the tumour, ascertained by the perception with a finger on the os uteri of a shock impressed upon the abdomen, is nearly decisive of a tumour being uterine, and the very feeble or absent impression of such shock. of its being ovarian. The exceptions are mainly those cases where adhesions have taken place, uniting the pelvic viscera closely together. Dr. Simpson of Edinburgh has recently proposed the use of a bougie for this purpose. It is to be introduced into the uterus, and then, he states, that by turning it one way, and pressing the tumour the other, it is quite possible to establish a distinction between the uterus and ovary in cases of ovarian disease. Or it might be possible that the direction taken by the bougie would establish the same fact.

Again, a careful examination per rectum and per vaginam will very often, even where the tumour is adherent, prove that there are two tumours, and their different density, or the comparative vividness of shocks communicated from the abdominal tumour, may justify the inference that one is the uterus and the other the ovary.

Lastly, the history of the disease may throw some light upon its nature. Uterine tumours are *generally* of slower growth, of smaller size, more dense to the touch, seldom attacked by inflammation, and rarely painful; and although none of these circumstances are conclusive alone, they may be very decisive in conjunction with other signs.

3. It may not be very difficult to come to a conclusion as to the existence of adhesions, though far from easy to estimate their extent. The mobility of the tumour, if it do not fill the entire abdomen, will generally decide the question; but when the disease attains an enormous volume, we can do little more than form a conjecture. There is a sort of rolling feel when a tolerably free ovarian tumour is moved, and a crepitus when adhesion has occurred, which is not easily mistaken; and a change of posture may afford additional information.

4. It is, of course, almost impossible to estimate the vascularity of an abdominal tumour. Occasionally we may distinguish with the finger the pulsation of an artery, and more than once I have ascertained the fact with the stethoscope. A careful examination should always be made with this instrument.

These hints may show at least the obscurity of the means of diagnosis, and perhaps aid a little in dispelling some of that obscurity. At all events it is certain that difficulty and doubt exist, or such mistakes would not have happened in the hands of careful men; and as these errors may be repeated, I would earnestly advise that when, on opening the abdomen, the tumour is found to be uterine, no attempt be made to remove it. The patient has a far better chance of recovery if the disease be untouched, and it is unlikely that any evil consequences will result from the tumour itself. It is a sad reproach that a patient should die, not of the operation, but in consequence of attempting that which was not originally contemplated.

*Conclusion.*—Even after the details I have given, it is very difficult to come to a definite and perfectly satisfactory conclusion, because, 1. we have not sufficiently accurate data to estimate the progress of the disease unaided by surgery. 2. The table quoted from Mr. Southam is clearly too limited to afford a fair average of the results of tapping, and it is not easy to obtain sufficient facts to enlarge it. 3. The cases in which ovariotomy has been performed are of such a mixed character, that it is impossible to select with fairness those cases in which the operation was demanded for the relief of urgent suffering, and suitable to the nature of the disease, without the appearance of partiality. And 4, from the obscurity of the diagnosis, it is too much, perhaps, to expect that our practice in future will be free from those drawbacks on the operation.

But bearing in mind these difficulties, and making allowance for those drawbacks, I think we may conclude that there are cases in which the operation would be justifiable; and on these grounds,—we find the general opinion is against the curability of the disease by medical means;—that after a time the patient will die from local disease or accident, or constitutional disturbance, and that meantime she suffers more or less inconvenience;—that tapping in almost all cases affords but temporary relief;—and that, as far as the limited statistics we have adduced are admissible as evidence, it is attended with great danger; i. e. 1 in 5 died of the first operation, and of twenty patients, fourteen (more than two-thirds) died within nine months of the first tapping; whilst of the entire number of those who underwent the operation of ovariotomy, about one-half have absolutely recovered so far.

We may add, that of those who died, some were in an unfavourable condition for any great operation, and many had no other hope of relief.

2. If we reject those cases in which the operation could not be completed—those in which it was unnecessary, and those when the patient laboured under organic disease, or a debilitated and broken constitution, the mortality is twelve in forty-two, or 1 in 3½. Even making allowance for the difficulty of diagnosis, it does appear to us that in future sufficient judgment may be exercised to reduce the proportion to something near this.

3. Again, if the operation were confined to cases of unilocular cysts without adhesions, or even to cases requiring the major operation where no adhesions exist, the results, according to our statistics, would be more favourable. At present it would seem desirable, if possible, to limit the operation to these cases.

4. Let it be observed, that so far we have canvassed the merits of the operation *on the recorded results*, not on the propriety or impropriety of it in any or all of the cases related; but it would be impossible to shut our eyes to the fact, that it has been sometimes performed without a due regard to the condition of the patient—to the necessity of an operation at all—or to the one in question being exactly adapted for the purpose.

To justify the operation in an individual case, the patient should be so far inconvenienced by the disease as to require surgical relief of some kind; and yet, on the other hand, she ought not to be in a condition which would prohibit other great surgical operations. In such cases the alternative is tapping or extirpation, and our judgment should be formed upon a careful estimate of the results of each.

Again, it is clear that no operation of this magnitude should be attempted when there is coincident organic disease of a serious character in other organs; nor have we sufficient evidence to justify an extension of the operation to other diseases than those of the ovaries.

5. As to the mode of operating, it appears to me, that it is better to commence with the small incision, and, if necessary, afterwards enlarge it. The great advantage of this plan appears to be, that after making the incision (in some sort an exploratory one,) if the sac, after being emptied, can be drawn out, we escape with the slighter risk; if there be obstacles, owing to solid matter, it can be enlarged without difficulty; and if these obstacles be such as to deter us from completing the operation, we can recede with much less danger to the patient; and this I think of vast importance, considering the present uncertainty of our diagnosis.

I have thus endeavoured to lay before the Society such information as I have been able to obtain concerning this important operation, without appearing as its advocate or its opponent, beyond what the statistical results will justify. These results are, I think, neither so favourable as some of its friends have represented, nor so discouraging as its opponents have asserted."

At a meeting of the *Royal Medical and Chirurgica Society of London*, held on the 25th of June last, Mr. Benjamin Phillips read a paper on this subject, based upon an examination of sixty-nine cases in which the operation was performed. "In fifty cases, a tumour was extracted; in fourteen cases, adhesions, or other circumstances, prevented its removal; and in five instances, no tumour was found. Of the cases in which the operation was completed, the tumour being extracted, thirty terminated favorably, and the patients recovered; in twenty instances, the termination was unfavorable, and the patients died. Of five cases in which no tumour was discovered, all recovered. Of the fourteen in which adhesions, or other circumstances, prevented the extraction of the tumour, eight recovered, and six died." It will thus be seen that Dr. Churchill and Mr. Phillips agree very nearly in their accounts of the statistics of this operation: there is also a very general concurrence between them on all the important points presented in the consideration of the subject of ovariotomy.

In the opinion of Mr. Phillips, "the preponderance of success is in favour of what is termed the minor operation, (as compared with the major) that is to say, an operation in which the incision is as small as is consistent with the easy removal of an *emptied* cyst, provided it be large enough for the convenient application of the ligature around the pedicle.

In the last number of the American Journal of the Medical Sciences, we have an account of a case operated on by Professor W. L. Atlee, of Lancaster, on the 29th of March last. The patient was 61 years old on the 26th of the preceding November—the incision extended from immediately below the umbilicus, in a straight line, to within one inch of the symphysis pubis. The uterus and right ovary were sound; the left, with its corresponding fallopian tube, was involved in the tumour which was removed. "The tumour, immediately after the operation, weighed ten and a quarter pounds; its greatest circumference being two feet nine inches; its smallest, two feet." The operation was performed with skill and tenderness, and the patient bore it well, but on the third of April (four days after the operation) she died, labouring under peritonitis. After death, "*a globular cyst, about the size of an orange*, was lifted up from the deepest part of the pelvis, and was found to be pendulous from the *right ovary*."

---

*Cyclopædia of Practical Medicine.*

Parts X. and XI. of this valuable work have appeared with their accustomed regularity. Part XII. will complete one half of the extensive undertaking.

